# Neural Predictors of Pharmacological Treatment Response in Obsessive-Compulsive Disorder: A Resting-State fMRI Study

**DOI:** 10.1192/j.eurpsy.2026.10245

**Published:** 2026-07-31

**Authors:** A. D. Costa, M. Picó-Pérez, P. Morgado

**Affiliations:** 1 Life and Health Sciences Research Institute (ICVS), School of Medicine, University of Minho; 2 ICVS/3B’s, PT Government Associate Laboratory; 3 2CA- Braga Clinical Academic Center, Braga, Portugal; 4 Departamento de Psicología Básica, Clínica y Psicobiología, Universitat Jaume I, Castelló de la Plana, Spain

## Abstract

**Introduction:**

Obsessive-compulsive disorder (OCD) is a chronic and disabling psychiatric condition for which pharmacological interventions are effective, yet clinical outcomes remain heterogeneous. Identifying neural predictors of treatment response could inform precision medicine approaches. Functional magnetic resonance imaging (fMRI), particularly resting-state functional connectivity (RS-FC), has emerged as a promising tool to characterize baseline neural markers associated with treatment outcomes.

**Objectives:**

This study aimed to investigate whether baseline RS-FC patterns predict clinical improvement in OCD patients undergoing pharmacological treatment.

**Methods:**

We analyzed a dataset comprising 27 adult OCD patients (15 females; mean age 33.5 ± 8.2 years) who underwent 3 months of pharmacological treatment. Clinical symptoms were evaluated using the Yale-Brown Obsessive Compulsive Scale (Y-BOCS) at baseline and post-treatment. Resting-state fMRI acquisitions were performed at baseline. Independent component analysis (ICA) was conducted to identify functional networks, and baseline RS-FC values were correlated with Y-BOCS difference (Post – Pre). Paired-sample t-tests assessed clinical outcomes.

**Results:**

Patients showed a significant reduction in global OCD symptoma tology following pharmacological intervention (Y-BOCS: Mean Pre = 31.82, SD = 4.83; Mean Post = 24.19, SD = 11.10; t(26) = 4.53, p < .001). Improvements were observed in both the Obsessions subscale (Mean Pre = 17.00, SD = 2.42; Mean Post = 12.41, SD = 5.44; t(26) = 4.85, p < .001) and the Compulsions subscale (Mean Pre = 14.82, SD = 4.10; Mean Post = 11.78, SD = 6.02; t(26) = 3.61, p = .001). Resting-state analysis revealed that decreased baseline FC in the right Executive Network (MNI coordinates: = x = -34, y= 53.9, z= 53.4, cluster extent = 25, p = .042), Salience Network (MNI coordinates: x= -70, y = -43.8, z= 5.02, cluster extent = 121, p = .013), and Cerebellar network (MNI coordinates: x= -64, y= 20, z= -32.7, cluster extent = 226, p = .040) were significantly associated with greater Y-BOCS symptom reduction, suggesting predictive relevance of these networks for pharmacological response. Effect sizes were medium-to-large across symptom reductions (Cohen’s d range: 0.70–0.95).

**Conclusions:**

Our findings indicate that baseline hyperconnectivity in the executive, salience and cerebellar networks predicts favorable response to pharmacological treatment in OCD. These results highlight the importance of balanced goal-directed behavior and action planning in the modulation of treatment outcomes and suggest that RS-FC markers may help stratify patients most likely to benefit from pharmacotherapy. Integrating neuroimaging predictors into clinical decision-making could support the development of personalized treatment strategies for OCD.

**Disclosure of Interest:**

A. D. Costa Grant / Research support from: PhD scholarship from the Foundation for Science and Technology (2022.09472. BD), M. Picó-Pérez: None Declared, P. Morgado Grant / Research support from: FLAD Science Award Mental Health, Consultant of: Angelini, AstraZeneca, Bial, Biogen, Janssen-Cilag, Lundbeck, Viatris

